# Notification of tuberculosis cases in India: Moving ahead in Revised National Tuberculosis Control Program

**DOI:** 10.3402/iee.v3i0.23006

**Published:** 2013-11-18

**Authors:** Saurabh RamBihariLal Shrivastava, Prateek Saurabh Shrivastava, Jegadeesh Ramasamy

**Affiliations:** Department of Community Medicine, Shri Sathya Sai Medical College and Research Institute, Kancheepuram, India

**Keywords:** tuberculosis, medical colleges, notification, private practitioners, Revised National Tuberculosis Control Program, India

## Abstract

Tuberculosis (TB) is currently the leading cause of death from a curable infectious disease accounting for 8.7 million new cases and 1.4 million deaths in the year 2011. From the year 2012, TB is a notifiable disease in India which means that all cases of TB diagnosed by any means has to be reported to the public health authorities. This would help policy makers to make rational decisions with regard to strengthening of existing infrastructure and scaling-up of TB control activities in the country. Employment of multiple measures directed towards different stakeholders can be strategically implemented to intensify and fast-track the process of TB notification. In conclusion, the Indian Government's decision to specify TB as a notifiable disease is a historical and a much awaited step in the TB control activities. However to obtain the desired results, program managers along with the health care workers have to work in an integrated and collaborative manner so that the burden of TB can be reduced in years to come.

Tuberculosis (TB) is currently the leading cause of death from a curable infectious disease accounting for 8.7 million new cases and 1.4 million deaths in the year 2011 (1). Out of the overall global burden, India alone has contributed to a quarter of all TB cases (1). Furthermore, it has been estimated that in India, everyday more than 40,000 persons will get infected with TB bacilli, more than 5,000 people will develop symptoms of TB disease and more than 900 people will die as the result of a TB infection (1, 2). Although the Revised National TB Control Program’ in India has adopted multiple strategies and innovations to decrease the number of infected individuals, the results have not been as good as expected (1). The results from the National Family Health Survey-3 revealed that the private sector was the preferred source of healthcare (70% of urban households and 63% of rural households) for patients diagnosed with TB. Thus, there is an indispensable need to involve both the public and private sectors in the national effort to counter the problem of TB (3).

Since 2012, TB is a notifiable disease in India, which means that all cases of TB diagnosed by any means (sputum examination/chest X-ray/other laboratory and radiological tests/clinical judgment) have to be reported to the public health authorities in a specified format. This initiative has been implemented so as to estimate the number of TB cases in the community more correctly. This would help policy makers to make rational and evidence-based planning with regard to strengthening of the existing infrastructure (viz. diagnostic aids/therapeutic/monitoring and supervision activities/human resources) and scaling-up of TB control activities in the country (4).

Employment of multiple measures directed toward different stakeholders can be strategically implemented to intensify and fast track the process of TB notification (4, 5). These targeted interventions can be directed toward the following stakeholders:

1. Private medical practitioners – conducting sensitization sessions in different aspects of TB diagnosis–treatment–notification; involving more and more private practitioners in a treatment adherence scheme; proactive collection of notification reports including nil-reports from private practitioners; and organizing monthly review meetings to monitor the process of notification.
2. Building partnerships with multiple agencies involved in the process of TB diagnosis, care and management – establishing linkages with professional associations like Indian Medical Association/Indian Pharmacist Association, and so on; registration of all laboratories/hospital/clinic/nursing home; and involving non-governmental/philanthropic organizations.
3. Medical colleges – organizing a monthly online review of the state task force committees with mandatory feedback; reinforcing about notification in academic teaching sessions; and encouragement of undergraduate and postgraduate students for adopting research work.
4. Community-based interventions – sensitization of general community/patients through different mass media aids; and organizing counseling sessions for patients by healthcare providers about the need of notification.
5. Innovations – adoption of online reporting; incentive/rewards to private practitioners for encouraging notification; and adoption of one district by every medical college to monitor and supervise notification activities.

In conclusion, the Indian Government's decision to specify TB as a notifiable disease is a historical and a much-awaited step in the TB control activities. However, to obtain the desired results, program managers along with the healthcare workers have to work in an integrated and collaborative manner so that the burden of TB can be reduced in years to come.

## Keypoints

1. Health authorities should be notified of any TB cases to assist the program managers in planning appropriate strategies to reduce the burden of the disease.
2. Every healthcare provider/clinical establishment run or managed by the Government/private sector should notify patients who are diagnosed with TB or who are initiated on anti-TB treatment.
3. The nodal public health authority should be notified in the prescribed format.
